# Perceptions and usage of selected fermented foods for feeding children aged 13-60 months in Tshwane, Gauteng Province

**DOI:** 10.11604/pamj.2020.36.293.25053

**Published:** 2020-08-17

**Authors:** Paul Kiprono Chelule, Sphiwe Madiba, Mathildah Mokgatle

**Affiliations:** 1 Department of Public Health, Sefako Makgatho Health Sciences University, Ga-Rankuwa, South Africa

**Keywords:** Fermented foods, perception, malnutrition, children

## Abstract

**Introduction:**

fermented indigenous foods are known to confer health and nutritional benefits to young children. However, perception and usage of these foods is not well understood. This study aimed to determine the perceptions and usage of fermented foods, by child caregivers, for children feeding in Gauteng Province, South Africa.

**Methods:**

a standard questionnaire was used to obtain data from child caregivers on the perceptions and usage of fermented foods to feed young children in Tshwane metro.

**Results:**

a total of 1248 child caregivers completed the questionnaires. Their age ranged from 15-65 years, 71.7% being single, with majority (74%) having attained secondary school education and unemployed (65%). Over 60% of children were fed sour milk (maas), sour porridge and yoghurt at a frequency of 1-3 times a week, with the feeding starting at the age of 6-12 months. Majority (59-74%) had positive perceptions on benefits of feeding children with fermented foods.

**Conclusion:**

there is a high acceptability of fermented foods for feeding children in this community. Further promotion of these foods may improve the frequency of their usage.

## Introduction

Despite effective health interventions, over 10 million children under the age of 5 years still die every year from preventable and treatable illnesses and in almost one-half of these cases, the illness is complicated by malnutrition [1]. Urban malnutrition is an increasing problem because about 72% of sub-Saharan Africa´s urban residents live in informal settlements characterised by limited livelihood opportunities and poor environmental sanitation [2]. The increase in the prevalence of malnutrition in sub-Saharan Africa means that the goal set to reduce the levels of undernutrition by any proportion may not be met [3]. Fermented foods and probiotics are indigenous foods that have been demonstrated to improve the nutritional value of food products such as maize and sorghum and thus confer nutritional and health benefits to both young children if used regularly as part of the daily dietary program [4-8]. For example, fermented foods have been demonstrated to reduce childhood diseases such as diarrhoea in hospitalised children, as is effective in eliminating the growth of diarrhoeal viruses and bacteria [5,9-13]. In other cultures, in sub-Saharan Africa, fermented foods have been used, not just for infant feeding, but for child weaning because of their improved food digestibility, desirable aroma, texture and flavor [14-16]. This, for example, can make the children eat more of the fermented than the unfermented products. Fermented food usage in food insecure communities, especially South Africa, is still poor [17]. Some published reports suggest that limited usage of fermented foods may be contributed by a number of factors, such as availability, lack of knowledge and perceptions [18,19]. Thus, the aim of this study was to assess the level of knowledge and perception of child caregivers on the use of fermented foods for feeding children aged between 13 and 60 months.

## Methods

This is a sub-study of a major cross-sectional study on the nutritional status of children under five in primary health facilities. The study participants were child caregivers of children aged between 13 and 60 months in Tshwane metro, South Africa. The caregivers were recruited from the well-baby clinics. Only consenting caregivers were recruited to participate in the study. Since this study data were obtained as part a bigger study, the detailed methodology is well described in a previously published article [20].

## Results

**Demographic profile of study of child caregivers:** in this study, the study participants were child caregivers of children aged between 13-60 months. A total of 1239 child caregivers participated in this study. Their age ranged from 16-65 years (mean of 30 years). Most of them were single (71.7%) and over half were in the 20-29 year old category, un-employed (73.2%) and largely relied on child grant support to bring up their children (67.3%) (Table 1). Majority (74%) had attained secondary school and grade 12 education and this could be the reason for their high unemployment rate.

**Table 1:** demographic profile of child caregivers (N=1239)

Variable	Description	Number	%
Age Category	15-19	52	4.2
	20-29	633	51.1
	30-39	395	31.9
	40-49	116	9.4
	50+	43	3.5
Marital status	Single	886	71.7
	Married	326	26.4
	Divorced/widowed	23	1.9
Educational status	Primary	82	6.6
	Secondary	370	29.9
	Grade 12	554	44.7
	Tertiary	233	18.8
Employment status	Not employed	907	73.2
	Employed	332	26.8
Child grant	No	391	32.7
	Yes	805	67.3

**Usage of fermented foods for child feeding:** in this study, child caregivers were asked for a list of foods that they used to feed their young children. The study findings show that over 60% (n=1239) of children were fed sour milk (maas) (64.3%), sour soft porridge (68.0%), which is usually made at home and yoghurt (89.1%) (Table 2). Sour milk (maas) and yoghurt are obtained from supermarkets and other commercial outlets as they are not easy to prepare at home. Ting and mageu were the least used fermented foods for children feeding (20.1% and 39.8% respectively). Ting is often prepared at home. When asked how often they used fermented for feeding, most of the child caregivers fed their children fermented foods at a frequency of at least 1-3 times a week (Table 3). Yoghurt, seconded by sour milk were the most frequent fermented foods given to the children (n-359 and 220 respectively). The frequency of feeding peaked at the first month and tapered towards 3 months. However, for some reason, 71.5% of caregivers fed sour porridge to their children every day of the week. No follow-up questions were asked to understand why. This may need to be further investigated. The data also shows that most caregivers (over 70%) initiated fermented food feeding to their children from 6-12 months, leaving a smaller proportion of caregivers to initiate feeding thereafter beyond the period of 12 months (Table 4). The higher proportion of caregivers initiating feeding at the 6-12 month category of children was similar in all selected fermented foods. Again, the child caregivers preferred sour porridge and yoghurt (90% and above) to initiate their fermented food feeding.

**Table 2:** usage of fermented foods for feeding children (N=1239)

Fermented food	Yes (%)	No (%)
Sour milk	797(64.3)	442(35.7)
Sour porridge	843(68.0)	396(32.0)
Ting	249(20.1)	990(79.9)
Yoghurt	1104(89.1)	135(10.9)
Mageu	493(39.8)	746(60.2)

**Table 3:** frequency of fermented food usage per week

Frequency per week	Sour milk	Sour porridge	Ting	Yoghurt	Mageu
1	220(35.7)	128(17.9)	71(47.3)	359(42.4)	189(64.5)
2	130(21.1)	52(7.3)	26(17.3)	164(19.4)	44(15.0)
3	66(10.7)	24(3.4)	6(4.0)	101(11.9)	14(4.8)
4	22(3.6)	-	2(1.3)	17(2.0)	2(7.0)
5	1(0.2)	-	-	6(0.7)	1(0.3)
6	-	-	-	-	-
7	178(28.9)	511(71.5)	45(30.0	199(23.5)	43(14.7)

**Table 4:** age of initiating fermented food feeding in children

Age (months)	Sour milk	Sour porridge	Ting	Yoghurt	Mageu
0-6	155(20.3)	571(70.0)	45(19.3)	344(32.7)	110(22.7)
7-12	446(58.5)	215(26.3)	123(52.8)	602(57.2)	262(54.1)
13-18	45(5.9)	7(0.86)	16(6.9)	21(2.0)	21(4.3)
19-24	79(10.3)	16(2.0)	21(9.0)	46(4.4)	53(11.0)
25-36	26(3.4)	5(0.6)	16(6.9)	37(3.5)	31(6.4)
37-48	9(1.2)	1(0.1)	9(3.9)	1(0.1)	6(1.2)
49-60	2(0.3)	1(0.1)	3(1.3)	1(0.1)	1(0.21)

**Caregivers perceptions of fermented foods for child feeding:** in this study, majority (59-74%) had positive perceptions of feeding children with fermented foods (Table 5). For example, over 60% of respondents correctly perceived that fermented foods were cheap to buy from commercial outlets, easy to prepare at home and had a long shelf-life and could not expire easily like other ready-to-eat foods. Most participants also perceived that fermented food was liked by the children (77.7%), was nutritious (81.6%) and not too sour for the children (67.6%). They also correctly perceived that fermented foods did not cause diarrhoea in children (85%). Although most of these caregivers were young (majority in the 20-29 year category), they acknowledged (77.2%) that fermented foods are not only meant to be consumed by old people, but by everybody else including the children.

**Table 5:** caregivers perceptions of fermented foods for child feeding

	Yes (%)	No (%)
Cheap to buy	850(68.8)	386(31.2)
Easy to prepare	1104(89.2)	133(10.8)
Long shelf life	741(60.1)	491(39.9)
Easy to store	483(39.2)	750(60.8)
Makes child eat more	732(59.1)	507(40.9)
Causes child diarrhoea	186(15.0)	1053(85.0)
Makes child loose weight	170(13.7)	1069(86.3)
Too sour for children	401(32.4)	838(67.6)
Child does not like it	276(22.3)	962(77.7)
It is not nutritious	228(18.4)	1011(81.6)
It is for old people	282(22.8%)	957(77.2%)

## Discussion

This study investigated the perceptions and usage of indigenous fermented foods to feed young children by their caregivers in a South African urban community. The caregivers comprised the mothers of the children in the study. This was an appropriate group for study participants as mothers are key role players in child feeding and food security in most African households [21]. Over half of the caregivers in the study were young mothers (20-29 years old) with most having achieved grade 12 level of education. As such, over 70% of them were unemployed and greatly depended and national child grants to raise their children. In this study, five indigenous fermented foods commonly consumed in South Africa (soft sour porridge, fermented milk, ting, mahewu and yoghurt) [6] were investigated for child feeding usage. Over 60% of caregivers in this study fed fermented foods to their under-5-year-old children. The most popular fermented foods for child feeding were fermented milk (maas), sour soft porridge and yoghurt at a frequency of 1-3 times a week. Ting was the least used among the five indigenous foods investigated in this study. Plausibly, this being a solid food, it is difficult for the children to ingest unlike the liquid forms of fermented foods (porridge and milk). These findings show that fermented foods are acceptable for children feeding in this community. This observation is evident in another published report from Zimbabwe, where over three quarters of caregivers fed their under-five-year-old children mahewu and yoghurt [22]. Reports from other countries in Africa also show that fermented foods are acceptable and safe to feed young children [12,23,24].

The most preferred child age for initiating fermented food feeding in this study ranged from 6-12 months. Although the reasons for initiating fermented food usage at this early age were not investigated, under normal circumstances, it is unusual for mothers to feed children fermented food in the first month of infant´s life. Most mothers usually initiate feeding around this period; at least around 6 months of child´s life. For example, a number of African nations reportedly initiated fermented food feeding to their children at the age of 4-15 months [12,22]. The most probable factors for initiating fermented food feeding in the early months of infant´s life could be due to the inability to initiate or continue with breastfeeding, for example, where the mother was HIV-infected (and thus, the usual six months exclusive breast feeding cannot be observed) or where the caregiver was not the biological mother of the child and may not be able to provide the breast milk [22]. The other possible reason the of fermented food usage for child-feeding could be the ease of availability, preparation and affordability, as most of the caregivers in this study were unemployed.

These factors are not unique to this study as other African researchers have reported the same. For example, in Zimbabwe, homemade mahewu was commonly used for child feeding, as the caregivers could not afford commercial infant milk formula [22]. The child caregivers were not following any dietary guidelines in the fermented food feeding in this study. However, published guidelines are readily available and would promote fermented food usage in children [25]. Most caregivers (over 60%) in this study had positive perceptions towards usage of fermented foods to feed their young. This perception is shared by Zimbabwean child caregivers as well [26]. This is contrary to other peddled rumours that fermented foods were not liked by children due to their sourness, could cause diarrhoea and weight loss [23]. It is also interesting to note the correct perception of caregivers that fermented foods are not just for older people but are beneficial to all the generations including their young. This perception is helpful in formulating strategies for the promotion of fermented food usage in African communities, particularly in urban area, where western style foods are more embraced.

**Limitations:** the fact that the study participants were urban dwellers, young and mostly unemployed, could be a major limitation of this study. Thus, the findings may not reflect the views of the all the rural populace, older and employed people. Thus, further studies should seek to bridge the identified gaps in the future.

**Ethical approval and consent to participate:** ethical clearance was sought from Sefako Makgatho University Research Ethics Committee (SMUREC). Permission to conduct the study was sought from the Tshwane district office and from the managers of the primary health care facilities. A written informed consent was obtained from the participants before the interviews.

## Conclusion

There is a high acceptability of fermented foods for feeding children in this community, based on the high level of knowledge and positive perceptions of childcare givers. Further promotion of these foods may improve the frequency of their usage.

### What is known about this topic

- Fermented foods and their products such as probiotics are suitable for feeding young children;
- There is limited usage of fermented foods to feed young children in South Africa and most global communities.

### What this study adds

- There is positive perception of fermented food and their usage to feed young children in the study setting;
- The participants know the nutritional value of fermented foods;
- Positive perception and knowledge make it easy to promote usage of fermented foods to feed young children in African communities.
